# Scientific Temperance

**Published:** 1894-03-03

**Authors:** 


### Studies in Applied Sociology.

### IV.—SCIENTIFIC TEMPERANCE.

Drunkenness is the most prominent vice of the Teutonic races. How can we cure it? Should we wait till the individual has reached maturity before warning him of the evil of strong drink, or shall we instruct him as a child? Shall we appeal only to his moral nature, or shall we show him the physical harm done by alcohol?

These questions agitate all reformers; but the answers of the most sincere and earnest vary much. In America several States have adopted the teaching of temperance in the public schools. The pioneer of the movement was Mrs. Mary Hunt, a member of the Women's Christian Temperance Union. Her desire was not, however, merely to point out the evils of drunkenness, but to make clear to children the physiological mischief wrought by drink. The task was not easy. In the first place there were no text-books on the subject. The average temperance book was insufficient; the average text-book of physiology contained almost nothing about the matter. Therefore it took some years to find the needed authors and publisher, and to obtain the literature required. But something more than books was necessary, and much of the instruction was given by lectures and experiments. The experiments were graduated, and were meant to show that alcohol impairs digestion, destroys the blood corpuscles, ruins the blood vessels, and paralyses the nerves. The processes of fermentation and distillation were also explained. The results of Mrs. Hunt's action have been that a large majority of the United States have made temperance education a compulsory part of their code. It is true that in some districts the law is not too severely enforced; but still we may assume that a vast majority of American children are learning the evils that attend the consumption of alcohol.

It is admitted, however, that in many schools the instruction is imperfectly comprehended. There is something to be desired, both as regards literature and pathology, in the following answer to a question in an examination paper on the effects of alcohol on the system: "When a man gets drinking rum, which contains alchoall, it goes to his brain, and makes him insenseable. Alchoall will quicken the pults, and the hart will beat faster than usual. Alchoall is poision, and will poision the blood." It is not for the sake of the spelling that we quote this *résumé* of the action of alcohol, but because the vagueness of the last sentence is reproduced more or less in almost all the answers. "Alcohol is a poison"—yes, sometimes. But a child who is told this, and sees anyone drink wine or spirits without falling down dead, becomes straightway a sceptic as to the dangers of alcoholism. The difficulty of teaching the pathological effects of alcohol without giving an erroneous impression of the immediate results is one of the objections raised to this method of instruction. Another is that the experiments often arouse curiosity instead of carrying conviction. One experiment Mrs. Hunt herself condemns—that, namely, which consists in tasting a small quantity of alcohol to prove that it has a burning taste and increases salivation. It is difficult to think that any sane temperance reformer would recommend this method of teaching. Curiosity will lead to the unauthorised repetition of the experiment, and the habit of drinking may be established instead of discouraged. It is as if the Spartans had made their own children, instead of their slaves, play the drunken helot. Indeed, in an article in the "Popular Educator" for December, 1891, it is recorded of one boy, the son of a publican, that after a course of this "temperance" instruction, in which he had learned the taste of alcohol, he boasted that he could now distinguish between all the different liquors in his father's store.

Another experiment we must unequivocally condemn. A frog is to be chloroformed, then the leg opened to show the nerves, and alcohol dropped on the nerves to show how it paralyses them. If this experiment is performed in school with a chloroformed frog, depend upon it that it will be repeated outside—with the frog unchloroformed! Anything in the nature of vivisection should be used with the greatest discretion, even with adults; to children it should never be shown at all. Children are instinctively cruel, through mere ignorance; their reasoning powers are undeveloped, and anything that bears the semblance of cruelty allowed within the school will form the excuse and justification for endless cruelty outside.

Yet, these defects being admitted, the teaching of temperance in schools seems to us to be a good thing. But surely, for children, it is better to point out the moral evil of indulgence. To teach anything scientifically implies the scientific mind—suspension of judgment, appreciation of conflicting evidence—in those taught as well as in the teacher. We fear it is as little likely to be found in the average School Board teacher as in his pupils. The evils of drunkenness, on the moral side, are so common that no child can be wholly ignorant of them, and it is legitimate also to point out the mischief it works on the body, but without the aid of experiments which themselves contain a moral danger. Moreover, on the scientific side alcoholism is a disease, no more to be cured by demonstrations of its folly than madness, of which it may be taken as a form. The drunkard knows that his brain and liver are becoming diseased through his indulgence, and knowing this he drinks again. It is not knowledge that he lacks, but strength of will. If he is to be saved it will be through hygienic care, judicious restraint, and proper treatment, added to wise moral influences.

It is on the moral side that the teacher can exercise the best influence. Every child cannot grasp the notion of paralysed nerve-centres and hob-nailed liver, but every child knows something of the misery wrought by drunkenness. The physical idea is too remote, while the moral result is near. The general idea of disease and death may be given, but any attempt to convey the effect of alcohol in making the blood-vessels brittle, or the like, will probably fail. Temperance would still take its due place in the teaching of hygiene, but only as ventilation, food, and bathing do, and in the same general way. To explain to children of eight or ten details of pathology, which, as our medical examiners would admit, many young men fail to comprehend, is a waste of effort, and may practically defeat the end desired. But with these limitations we would cordially recommend the teaching of temperance in our national schools. It should be insisted, however, that any teacher who should speak of the evils of alcoholism should not indulge in wholesale denunciation of alcohol itself, which has its virtues as well as its dangers.

